# Single-Sensor Impact Source Localization Method for Anisotropic Glass Fiber Composite Wind Turbine Blades

**DOI:** 10.3390/s25144466

**Published:** 2025-07-17

**Authors:** Liping Huang, Kai Lu, Liang Zeng

**Affiliations:** 1 School of Mechanical Engineering, Xi’an University of Science and Technology, Xi’an 710054, China; lipinghuang@xust.edu.cn (L.H.); kailu@stu.xust.edu.cn (K.L.); 2 School of Mechanical Engineering, Xi’an Jiaotong University, Xi’an 710049, China

**Keywords:** acoustic emission, source localization, deep learning, wind turbine blades, single sensor

## Abstract

The wind turbine blade is subject to multi-source impacts, such as bird strikes, lightning strikes, and hail, throughout its extended service. Accurate localization of those impact sources is a key technical link in structural health monitoring of the wind turbine blade. In this paper, a single-sensor impact source localization method is proposed. Capitalizing on deep learning frameworks, this method innovatively transforms the impact source localization problem into a classification task, thereby eliminating the need for anisotropy compensation and correction required by conventional localization algorithms. Furthermore, it leverages the inherent coding effects of the blade’s material and geometric anisotropy on impact sources originating from different positions, enabling localization using only a single sensor. Experimental results show that the method has a high localization accuracy of 96.9% under single-sensor conditions, which significantly reduces the cost compared to the traditional multi-sensor array scheme. This study provides a cost-effective solution for real-time detection of wind turbine blade impact events.

## 1. Introduction

The turbine blades serve as the key component for efficient wind energy capturing. In the pursuit of cost reduction and efficiency enhancement, the design of blades has evolved to adopt a larger and lighter configuration, thereby concomitantly increasing their complexity [1,2]. At present, the construction cost of blades constitutes approximately 20% of the total cost of a wind turbine. Meanwhile, the blade failure accounts for 30–40% of total wind turbine failures [3,4,5]. This is attributed to the complex structural characteristics and harsh operating environments (e.g., lightning strikes, hailstorms, and foreign body impacts) of wind turbine blades. Such environments render blades susceptible to non-structural damage, including coating failure and leading-edge corrosion, as well as structural damage, such as composite layer failure, adhesion failure, and tip/root damage [6,7,8]. Therefore, it is imperative to accurately localize the impact source and designate damage-sensitive areas to ensure the safe operation of wind turbine blades.

For impact source localization, strain sensing methods are constrained by inherent bandwidth limitations, impeding effective analysis of high-frequency impact transient responses [9,10]. Ultrasonic detection methods face challenges in the real-time localization of impact sources due to the inherent conflict between preset excitation modes and the random characteristics of impact events [11,12,13]. In contrast, acoustic emission (AE) technology may be a promising candidate, as it can timely capture the stress waves in a wide frequency range and accurately localize the impact source [14,15,16,17,18,19]. However, the AE technique faces the challenge that its trigger threshold mechanism results in the loss of absolute signal delay information. This has led to the common adoption of the time difference of arrival (TDOA) method for signals received from several sensors at different spatial locations in existing studies [20,21,22,23]. This method establishes hyperbolic equations by converting the time difference into distance difference to finally obtain the source location. However, it is noted that a known wave velocity in all directions is required.

Consequently, a number of enhanced methodologies have been proposed. Al-Jumaili et al. [24] employed the Delta T method to create a training map using artificial sources in a specific region, and determined the AE source location through a clustering algorithm and a “minimum difference” method. Bhandari et al. [25] constructed a model based on empirical data that leverages a response surface developed from critical parameters (e.g., velocity variation, attenuation rate, distance from the sensor to the source, and fiber orientation) to update velocity data. Jones et al. [26] proposed a Bayesian source localization strategy, in which the source position is learned with respect to the corresponding arrival times of multiple sensors. Zhou et al. [27] employed 16 piezoelectric sensors and proposed a constrained total least squares (CTLS) method for jointly estimating the acoustic emission source and the wave velocity using TDOA measurements. Sikdar et al. [28] utilized eight AE sensors to obtain the response and dispersion characteristics of acoustic emission in a sandwich composite structure. Those methods can yield precise localization outcomes in anisotropic mediums; however, the deployment of multiple sensors concomitantly elevates the structural health inspection system’s complexity and expense.

Deep learning has significant advantages in learning the propagation characteristics of acoustic waves in non-uniform media through multi-layer convolutional neural networks [29,30,31]. It could eliminate the need for manual definition of acoustic velocity tensor parameters in anisotropic materials, and further, its hierarchical structure progressively extracts complex information from raw AE signals [32,33,34]. Thus, it may provide a promising candidate for impact source localization in complex mediums, e.g., wind turbine blades.

In this paper, a deep learning-based single-sensor impact localization method is proposed for the objective of reducing the sensor elements while retaining the localization precision. This method is distinct from prior techniques that necessitate the reduction or elimination of anisotropy through velocity compensation. Its innovation lies in the transformation of geometry complexity and material anisotropy characteristics into positioning advantages, thereby enabling single-sensor impact localization. The rest of the paper is organized as follows: Section 2 introduces the methodology. Section 3 describes the experimental setup in detail, including wind turbine blade spar specimens, sensor configuration, data acquisition, and experimental signal processing. Section 4 presents the localization results, error analysis, and comparative studies with alternative feature combinations. Finally, Section 5 draws the conclusions.

## 2. Methodology

**Figure 1:**
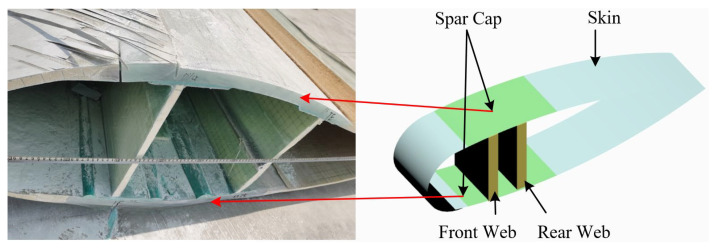
Wind turbine blade structures.

As demonstrated in Figure 1, the wind turbine blade principally consists of the skin, the spar, and the webs. The spar constitutes the primary component that provides support to the wind turbine blade, with its principal function being to resist wind loads and serve as the principal load-bearing structure. It is a pivotal component in determining the overall performance and stability of the blade. Therefore, subsequent studies will focus on the spar of the wind turbine blade.

**Figure 2:**
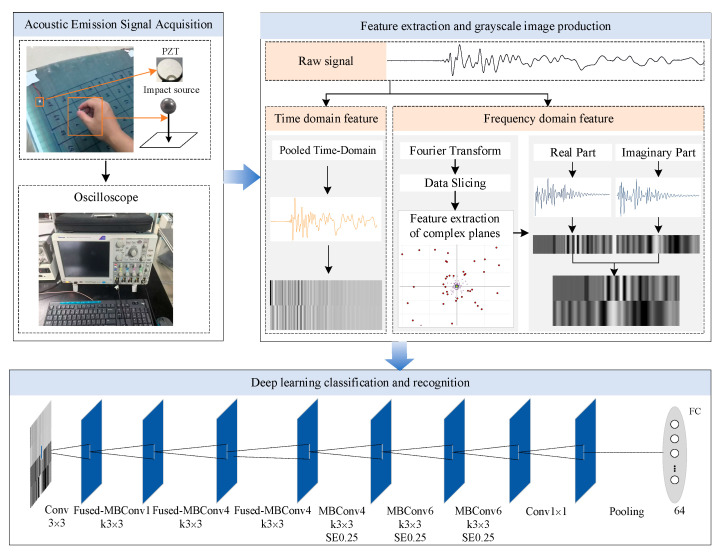
Overall framework of the proposed impact source localization method.

The spar is distinguished by its variable cross-sections, anisotropy, and material inhomogeneity, which collectively engender a high degree of complexity in the propagation of acoustic emission signals within it. These structural characteristics result in the manifestation of diversified propagation characteristics when AE signals transmit from disparate locations to the sensor. It is evident that disparities in propagation patterns are ultimately reflected in variations in signal features. The extraction of these signal features, along with the establishment of their correlation laws with spatial location, has the potential to facilitate impact localization with a single sensor and thereby constitute a possible alternative to traditional sensor array localization methods. To achieve that, this section proposes a methodology, including two major parts, i.e., feature matrix construction and impact source localization via EfficientNetV2-S, which transforms the impact source localization problem into a classification problem. Figure 2 gives the general progression of the method.

### 2.1. Feature Matrix Construction

#### 2.1.1. Frequency-Domain Feature Selection

Firstly, the Fourier transform (FT) is employed, converting the original waveform into frequency domain,

(1) $$F\left(\omega \right)=\int_{-\infty }^{\infty }f\left(t\right)e^{-i\omega t}dt,$$

where *f*(*t*) is the AE signal. Either the real and imaginary parts of *F*(*ω*) or the amplitude and phase of that are able to uniquely and completely characterize the original signal, thereby ensuring that the information is retained without distortion. As the phase is quite fragile to measurement noises, the real and imaginary parts are taken as frequency-domain features. However, those features are extremely redundant. If they are all used for the model training, it is very time-consuming and unnecessary. Therefore, the key problem lies in how to effectively compress the data volume of frequency-domain features while preserving sufficient information for impact localization.

To address this issue, the Kneedle algorithm is introduced, and its steps are given as follows [35,36]:

(1) *F*(ω) is rearranged in accordance with the amplitude value of each frequency from high to low, giving rise to the reordered spectrogram *F_s_*.
(2) Smoothing splines are employed to retain the waveform of the reordered spectrogram as

(2) $$F_{s}^{\prime }=\left\{\left(x_{i}^{\prime },y_{i}^{\prime }\right)\in {\mathbb{R}}^{2}\left|\;x_{i}^{\prime },y_{i}^{\prime }\geq 0\right.\right\},$$

where $F_{s}^{\prime }$ denotes the dataset fit to a smoothing spline of the frequency point $x_{s}^{\prime }$ and the corresponding amplitude value $y_{s}^{\prime }$.

(3) The points of the smooth curve are normalized as

(3) $$\begin{array}{l} D_{sn}=\left\{\left(x_{sn_{i}},y_{sn_{i}}\right)\right\},\;where\\ x_{sn_{i}}=\frac{\left(x_{i}^{\prime }-\min\left\{x^{\prime }\right\}\right)}{\left(\max\left\{x^{\prime }\right\}-\min\left\{x^{\prime }\right\}\right)},\;y_{sn_{i}}=\frac{\left(y_{i}^{\prime }-\min\left\{y^{\prime }\right\}\right)}{\left(\max\left\{y^{\prime }\right\}-\min\left\{y^{\prime }\right\}\right)}. \end{array}$$

where *D_sn_* denotes the normalized smoothed data.

(4) The difference curve (i.e., *D_d_*) between the smoothed curve *D_sn_* and the line connecting its first and last points is calculated, i.e.,

(4) $$\begin{array}{l} D_{d}=\left\{\left(x_{d_{i}},y_{d_{i}}\right)\right\},\;where\\ x_{d_{i}}=x_{sn_{i}},y_{d_{i}}=1-y_{sn_{i}}-x_{sn_{i}}. \end{array},$$

(5) Local maximum points are identified in the difference curve that correspond to candidate knee points where the original curve converges to a level;

(5) $$\begin{array}{l} D_{lmx}=\left\{\left(x_{lmx_{i}},y_{lmx_{i}}\right)\right\},\;where\\ x_{lmx_{i}}=x_{d_{i}},\\ y_{lmx_{i}}=y_{d_{i}}\;\left|\;y_{d_{i-1}}<\right.y_{d_{i}},\;y_{d_{i}+1}<y_{d_{i}}. \end{array},$$

(6) The dynamic threshold is set upon the sensitivity parameter, designated *S*, which serves to ascertain whether the knee point has been attained;

(6) $$T_{lmx_{i}}=y_{lmx_{i}}-\frac{S}{n-1}\sum_{i=1}^{n-1}\left(x_{sn_{i+1}}-x_{sn_{i}}\right),$$

where $T_{lmx_{i}}$ denotes the threshold for local maxima in the difference curve.

(7) In the event of a continuous decrease in the data until the next local maximum of the difference curve is reached and the data falls below the threshold of the current local maximum, the current local maximum point is recognized as the knee point, as illustrated in Equation (7). Conversely, if the difference increases, the threshold is reset to 0 and the next local maximum point is sought.

(7) $$Kneedle=\left\{D_{lmx_{i}}|T_{lmx_{i}}\leq y_{d_{j}},d_{j}=lmx_{i},lmx_{i}+1,\ldots ,lmx_{i+1}-1\right\},$$

The numerical value of *K* denotes the serial number of the relevant Kneedle point in the original curve. The frequency point exhibiting an amplitude greater than that at the corresponding frequency of *K* is determined as *G*. The frequency components preceding *G* are designated as the primary frequency components. This is done by extracting the real and imaginary parts of the previously obtained frequency. This process enables the compression of frequency data. Specifically, the extracted primary frequency is designated as *ω_p_*, and the corresponding frequency of the Kneedle point is denoted as *ω_k_*.

(8) Fp=ReFωp,ImFωp  absFωp≥absFωk.

#### 2.1.2. Time-Domain Feature Selection

Similarly to the frequency-domain features, the original time-domain signals also contain a substantial number of discrete data points, and direct processing results in an exponential increase in computational complexity. To address this issue, time-domain one-dimensional pooling is applied to compress the original long sequence signal into a compact sequence. It could reduce the data dimensionality by statistical aggregation (e.g., mean, maximum, etc.) of local regions of the signal, while preserving the main time-domain features of the signal (e.g., arrival time, waveform characteristics, etc.) [37].

Mean pooling is able to better reflect the overall trend of the region by averaging the local area, while having a better smoothing effect [38]. Its mathematical expression is

(9) $$T_{p_{i}}=\frac{1}{m}\sum_{\left(i-1\right)s+1}^{i\cdot s}f\left(t\right),i=1,2,\ldots ,H,$$

where *T_p_* is the pooled time-domain signal; *s* is the pooling step size; *m* is the pooling window size; *f*(*t*) is the original time-domain signal.

*H* is the length of the pooled signal and *N* is the length of the signal. The calculation formula is,

(10) $$H=\left[\frac{N-m}{s}\right]+1,$$

The implementation of the average pooling method results in the weakening of the transient information contained within the acoustic emission signal. However, the integration of the frequency-domain characteristics can facilitate the consideration of both global trends and local mutations. This, in turn, provides the feasibility of real-time localization of the impact source based on acoustic emission.

#### 2.1.3. Feature Matrix Construction and Grayscale Map Transformation

In the construction of the feature matrix, a multi-feature fusion method is employed in order to ensure full utilization of the frequency- and time-domain information of the signal. Specifically, the real parts of frequency-domain features determined by (8) are used as the first part of the matrix, the imaginary ones are used as the second part, and the time-domain one-dimensional average pooling of the signal is taken as the third part. It is ensured that the weights of the three parts are consistent. This construction method has been demonstrated to preserve the frequency-domain characteristics of the signal (through the real and imaginary parts) whilst simultaneously extracting the main trends in the time domain (through the average pooling operation). The formation of a comprehensive feature matrix provides a rich information base for the subsequent generation of a grayscale map and neural network classification.

In this study, the grayscale map is constructed for visualizing one-dimensional signal feature data and extracting features as an input. The generation process contains two key steps.

Firstly, the 1D signal feature data after multi-feature fusion is converted into matrix form. Specifically, the matrix *M* is constructed as follows:

(11) M=ReFωp, ImFωp, Tp.

Secondly, the matrix element values are linearly mapped to the 0–255 grayscale interval by the grayscale mapping algorithm.

(12) $${M^{\prime }}_{i,j}=\frac{M_{i,j}-M_{\min}}{M_{\max}-M_{\min}}\times 255,$$

where *M_min_* and *M_max_* are the minimum and maximum values in the matrix, respectively, and *M’* is the normalized matrix.

The matrix *M’* is then mapped to a grayscale image, where each element of the matrix corresponds to a pixel value in the resulting grayscale map. Subsequently, this grayscale image is utilized as input to the deep learning network.

### 2.2. Impact Source Localization via EfficientNetV2-S

#### 2.2.1. Deep Learning Model Selection

The EfficientNet model is selected for impact source localization, whose core feature is the combination of compound scaling and depthwise separable convolution (DW Conv), thereby enhancing the model’s performance through lightweight design and efficient feature extraction [39]. In this section, the mechanism of DW efficient convolution and its synergy with composite scaling will be briefly explained.

The objective of composite scaling is to optimize the scaling of the three dimensions of the network in a synergistic manner. Depth is defined as the number of network layers, and increasing the depth would enhance the nonlinear representation of the network. Width is defined as the number of channels per layer, and increasing the width improves the richness of the feature maps but significantly increases the computational effort. Resolution is defined as the size of the input image, and increasing the resolution helps to capture detailed features but consumes more memory. The three dimensions should be scaled concurrently at a constant ratio to attain a balance between optimal performance and computational efficiency [40]. Its mathematical form is

(13) $$Depth:d=\alpha^{\phi },Width:w=\beta^{\phi },Resolution:r=\gamma^{\phi }$$

where *α*, *β*, *γ* are the base scaling factors for each dimension and *ϕ* is the global scaling factor. The optimal scaling *α*⋅*β*^2^*⋅γ*^2^ ≈ 2 is determined by grid search to ensure that the amount of computation grows linearly with *ϕ*.

Due to the limited arithmetic power, the implementation of the composite scaling approach requires an efficient convolution approach. Depth separable convolution extracts spatial features by depth convolution and extracts channel features by point-by-point convolution, thereby reducing the computation significantly. It is divided into two steps, i.e.,:

Step 1. Depthwise Convolution

Each input channel is spatially filtered independently, using a single-channel convolution kernel with the same number of input channels. The computation is *H × W × C × L × L*, where *H × W* is the feature map resolution, *C* is the number of input channels, and *L* is the convolution kernel size.

Step 2. Pointwise Convolution

The number of output channels is adjusted using a 1 × 1 convolutional kernel cross-channel fusion features. The computation is *H* × *W* × *C* × *C*′, where C′ is the number of output channels. The standard convolution is computed as *H* × *W* × *C* × *C*′ × *L*^2^, and the total computation of the depth-separable convolution is only 1/*L*^2^ + 1/*C*′ that of the standard convolution.

EfficientNetV2-S, as a variant of the second generation of the EfficientNet series, has been shown to optimize the training speed by reducing redundant modules while maintaining performance [39]. Its lightweight design of the model effectively avoids the overfitting risk of traditional depth models in small-sample scenarios. Furthermore, the multi-scale feature fusion mechanism of the model can adaptively capture the complex spatial and temporal correlation features derived from the strong anisotropy of wind turbine blade materials during the propagation process of AE waves.

#### 2.2.2. Impact Source Localization

To achieve impact source localization, the method proposed in this paper integrates signal feature extraction with deep learning classification models. The following key steps are to be noted:

(1) Acoustic Emission Signal Acquisition: Capturing the shock-event-triggered wide-frequency-domain stress wave signals via a single PZT sensor;
(2) Feature Extraction: Involving the use of Fourier transform to extract the real and imaginary components in frequency domain, and the compression of time-domain signals by average pooling to preserve their arrival delays and waveform features;
(3) Grayscale Map Transformation: Involving the integration of frequency-domain and time-domain features into a two-dimensional matrix, and then that matrix is normalized and mapped to a 128 × 128 grayscale image;
(4) Model Training and Classification: The grayscale map is to be entered into the EfficientNetV2-S model, with the resultant probability distribution of 64 regions being outputted through the Softmax layer. This completes the classification and identification of the location of the impact source.

**Figure 3:**
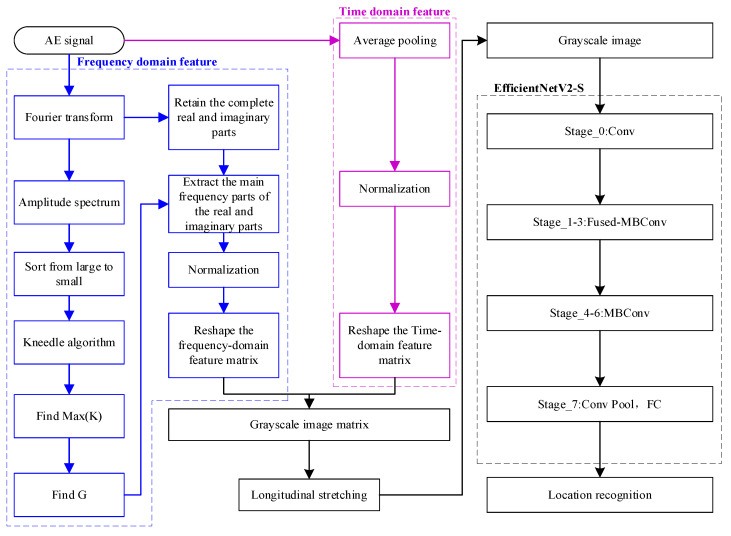
Detailed steps of impact source location.

The process establishes the mapping relationship between acoustic emission signal features and spatial location through a data-driven approach, thus avoiding the dependence on wave velocity and medium isotropy of traditional methods. As illustrated in Figure 3, the complete flow from the original signal to the result of the localization is visualized, thus providing a framework for subsequent experimental validation.

## 3. Experiments

### 3.1. Experimental Setup

**Figure 4:**
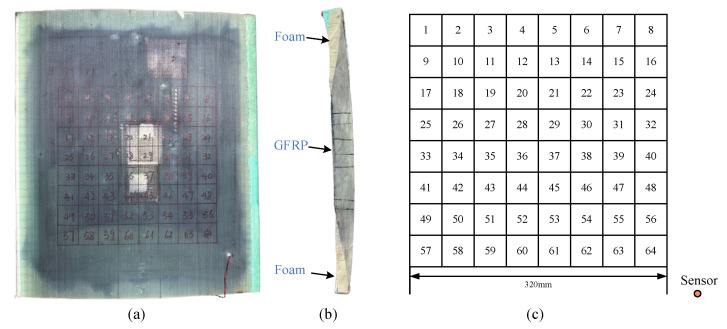
Experimental setups: (**a**) front of specimen, (**b**) cross-section, and (**c**) schematic diagram of grid division.

To verify the capability of the proposed single-sensor impact source localization method, experiments are conducted on a wind turbine blade spar specimen. As shown in Figure 4, the spar constitutes a heterogeneous structure composed of glass-fiber reinforced plastic (GFRP) and foam (i.e., Figure 4b). The specimen’s chord length is measured as 600 mm, the width is 570 mm, while the cross-section varies continuously from one end to the other, e.g., the thickness increases from 34 mm to 40 mm. The monitoring area is subdivided into 64 regions; each has the dimensions of 40 mm × 40 mm (see Figure 4c). The impact is simulated by Φ5 mm steel ball falling freely. The AE signals are captured by a single PZT and recorded by a Tektronix DPO5054B oscilloscope (Tektronix, Inc., Beaverton, OR, United States) with a sampling frequency of 2 MHz and a data length of 10,000 points. Each region is subjected to 15 random impacts of the ball. A total of 960 experimental signals are generated by traversing all 64 regions.

### 3.2. Experimental Data Processing

**Figure 5:**
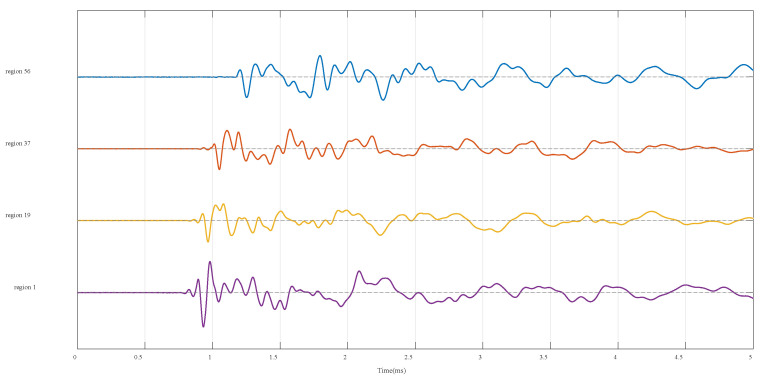
Time-domain waveforms of AE signals collected from different regions.

For illustration, Figure 5 displays the time-domain waveforms of AE signals as the ball drops into regions 1, 19, 37, and 56, respectively. To demonstrate the process of feature extraction, the AE signal of region 56 is taken as an example.

**Figure 6:**
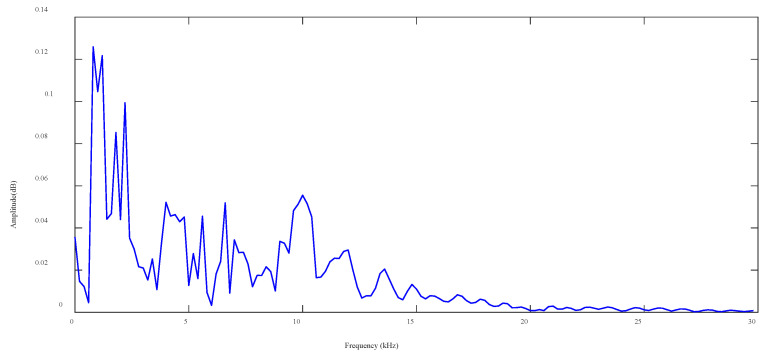
Frequency spectrum of AE signal in region 56.

Firstly, Fast Fourier Transform (FFT) is employed, converting the time-domain signal into frequency domain. Then, the amplitude spectrum is calculated and displayed in Figure 6.

Kneedle algorithm (Section 2.1) is utilized for automatic identification of the demarcation point between primary and secondary frequencies in the amplitude spectrum, e.g., Figure 6. This process involves the retention of the primary frequencies and the compression of data. As the value of sensitivity *S* determines the *K* value obtained by the Kneedle algorithm, it is essential to determine *S* in advance. If *S* is set too low, the algorithm exhibits excessive sensitivity, resulting in high variance. As *S* increases, the algorithm becomes more conservative. The choice of *S* has been extensively discussed in [35]. Based on its findings, Kneedle algorithm achieves the best results as *S* is set to 1. Therefore, this parameter value is also taken for subsequent analyses of this study.

**Figure 7:**
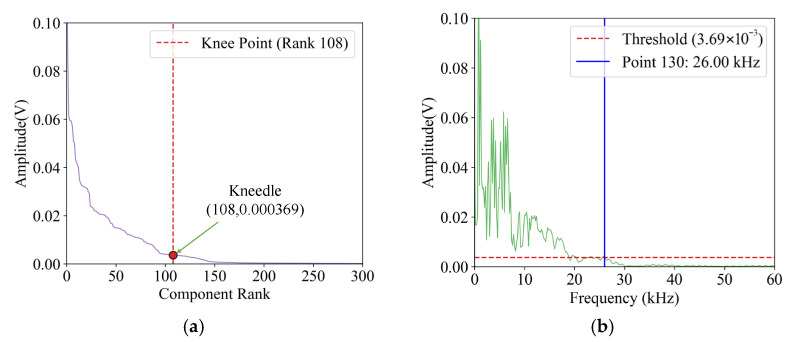
Amplitude ranking distribution of region 56: (**a**) amplitude plots sorted from largest to smallest, (**b**) feature point selection.

Subsequently, the amplitude spectrum *A*(*f*) is sorted by amplitude from high to low in order to obtain the rearranged amplitude list *A*(*f_r_*) and the corresponding frequency list *f_r_*; see Figure 7a. After that, the data is smoothed and then normalized by using Equation (3) to ensure that the horizontal and vertical coordinates of *A*(*f_r_*) are in the range of 0–1. Substituting *A*(*f_r_*) and *f_r_* into Equation (4), the difference curve could be constructed. Finally, Equations (5)–(7) are used to calculate the knee point, with the sensitivity *S* taking 1. The result is displayed in Figure 7a, where the knee point appears at the 108nd point in the sorted amplitude spectrum, i.e., *A*(*f_r_*) = 0.00369 V and *K* = 108. Subsequently, the maximum frequency point with an amplitude of 0.00369 V is identified in Figure 7b, and the *G* index is determined as 130.

**Figure 8:**
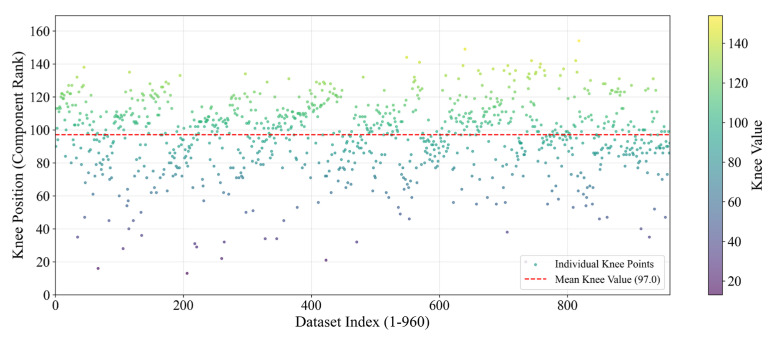
Knee point distribution across all 960 experimental signals (*S* = 1).

By the same way, the *K* values for all 960 experimental signals are obtained; see Figure 8. The mean value of *K* (i.e., 97) and the corresponding *G* (i.e., 102) is taken for the extraction of the primary frequency components. The real part Re(*f_k*) and the imaginary part Im(*f_k*) of the complex spectrum corresponding to each primary frequency are saved separately.

**Figure 9:**
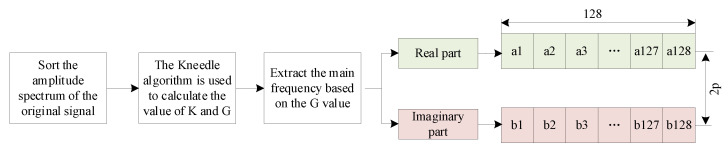
Frequency-domain feature extraction process.

In accordance with the *G* value (i.e., 102), the input image size of EfficientnetV2-s model takes 128 × 128, with the smallest integer multiple of 128 being selected for *p* = 1 as 128 × 1 > 102. Subsequently, the real parts of the 102 primary frequency points are arranged into one row (128 points per row), padding the remaining positions with zeros. The imaginary parts are then treated in the same way. This constitutes a 2 × 128 frequency-domain feature matrix. The sequence of events in this section is illustrated in Figure 9.

**Figure 10:**
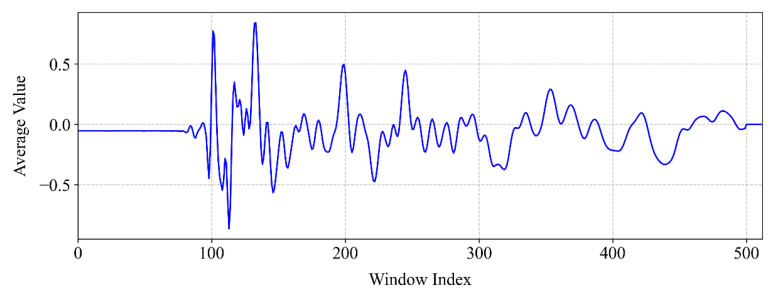
The time-domain signal related to region 56 after one-dimensional average pooling.

For time-domain feature extraction, the average pooling method is applied to the original time-domain signal with the step length and window length both taking 20. As the original AE signal contains 10,000 data points, the length of the pooled signal could be calculated from Equation (10) as 500. In this case, the length of the time-domain features, *L_t_*, takes 128 × 4 = 512 > 500, with 12 additional zeros appended to its end; see Figure 10. The time-domain feature length is then reorganized into a 4 × 128 feature matrix.

**Figure 11:**
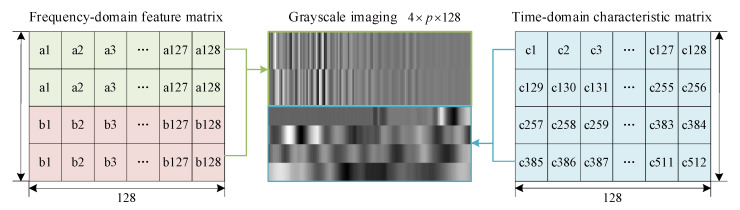
Feature matrix to grayscale map.

To ensure the consistency of the weights of frequency-domain features and time-domain features, it is necessary to ensure that the length of the frequency-domain features is equivalent to that of the time-domain features. Hence, the real and imaginary parts in Figure 9 are duplicated separately, thereby expanding the frequency-domain feature matrix (i.e., the left part of Figure 11). After that, the frequency-domain feature matrix and the time-domain feature matrix are spliced vertically into an 8 × 128 feature matrix and stretched vertically so that the grayscale image has a size of 128 × 128, as illustrated in Figure 11. That grayscale image is the input to the neural network, i.e., EfficientnetV2-S.

## 4. Results and Discussions

### 4.1. Impact Source Localization via the Proposed Method

**Figure 12:**
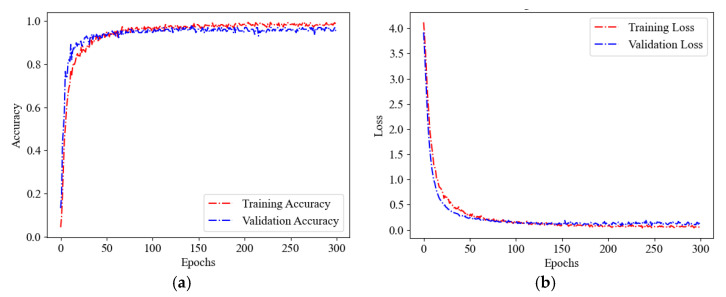
Model training process: (**a**) correctness curve, (**b**) loss curve.

The 960 experimental signals collected from the experiments are divided into training, validation, and test sets in a ratio of 11:2:2, while ensuring that the number of categories is consistent across each dataset. The grayscale images within the training set are utilized for EfficientnetV2-S model training, with the parameters for that model listed in Table 1. An incremental learning strategy is employed which is capable of adapting the regularization according to the dimensions of the image. During the initial stage of straining, the network is first exposed to images of reduced size and with weak regularization, then the image size is increased and stronger regularization is introduced. The initial model parameters are as follows: the image size is set to 0, the random augmentation is set to 5, the Mixup alpha is set to 0, the dropout rate is set to 0.1, the learning rate is set to 0.001, and the RMSProp optimizer has decay of 0.9 and momentum of 0.9. The model has been trained for a total of 300 epochs, with a batch size of 32 and a total batch size of 832. For illustration, Figure 12 gives the model training process as the grayscale image in Figure 11 is taken for input.

To discuss the effects of time- and frequency-domain features on localization performance, four feature combination schemes are considered. Specifically, in scenarios 1 and 2, the construction of the grayscale map is constructed by the real part and imaginary part in frequency domain, respectively. In scenario 3, both real and imaginary parts are used, thereby preserving the frequency features. In scenario 4, besides the frequency features, the time-domain average pooled features are also incorporated. To ensure fairness, except for the features used, the training set division, model architecture, and hyperparameter settings are all the same. The comparison of performance under the four feature combination schemes is presented in Table 2. For scenarios 1 and 2, the accuracies of the test sets are only 68.8% and 75.8%, respectively, indicating that it is difficult to capture the spatial coding characteristics of the signal by using either the real or imaginary part alone. In scenario 3, the fusion of real and imaginary parts has been demonstrated to obviously enhance the accuracy of the test set to 86.7%. This outcome validates the efficacy of integral frequency-domain features in enhancing the model’s capacity to differentiate different regions. The incorporation of time-domain pooling features in scenario 4 enhances the accuracy of the test set to 96.9%, thereby demonstrating that the supplementary time-domain features augment the model’s sensitivity to impact source locations. In addition, statistics are conducted on cases of inaccurate positioning and the results are also listed in Table 2. *X_error_* and *Y_error_* denote the localization errors of the inaccurate cases in the *x*-coordinate and *y*-coordinate, respectively. It can be seen that most of the misclassified cases in scenario 4 locate the impact source into its adjacent region, i.e., *X_error_ ≤* 1 and *Y_error_* ≤ 1.

### 4.2. Error Analysis

**Figure 13:**
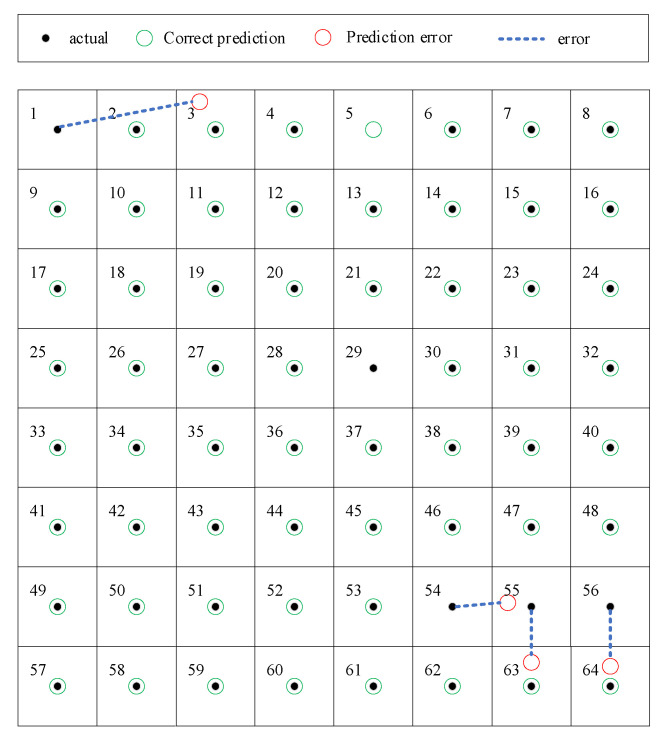
Localization results of the four misclassified samples.

**Figure 14:**
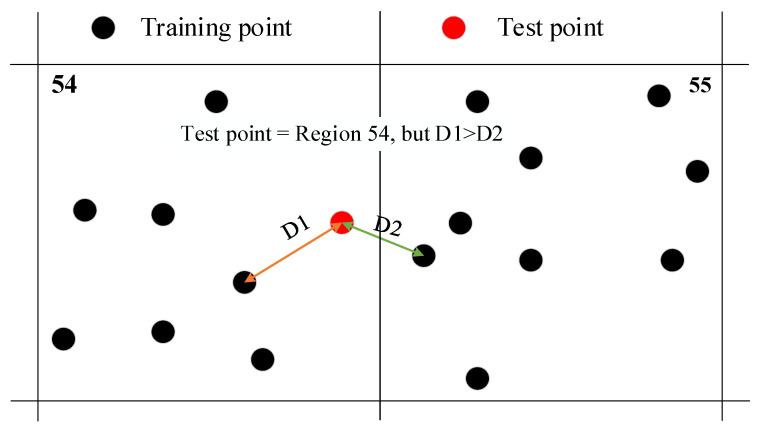
An example illustrates the reason for misclassification.

In this section, only the fourth scheme is considered. In that case, among the 128 samples in the test set, only 4 are misclassified, achieving an impact localization accuracy of 96.9%. Figure 13 shows the localization results of all 128 samples. In this figure, black dots represent the true class labels of the samples. Green circles centered on these dots indicate classifications correctly predicted by the proposed method. The four red circles offset from their respective black dots (i.e., those connected to red circles via blue dashed lines) denote misclassified samples, with their positions identifying the algorithm-predicted class regions. Table 3 lists the related localization errors in *x*-coordinate and *y*-coordinate. Three of them are positioned adjacent to the correct positions, resulting in a maximum deviation of one grid unit in either the x or y coordinate. The reason for this misclassification may be illustrated by Figure 14. In the experiment, although the monitoring area was divided into 64 regions, the landing location of the ball within each region is random. Consequently, it is possible that during one trial in the test set, even though the ball lands within region 54, its landing point might actually be closer to a landing point from a trial in the training set that occurred in region 55. This could cause its signal characteristics to be classified as belonging to region 55.

It is noteworthy that all localization results exhibiting only one grid error may be regarded as accurate. The reason may be twofold. Firstly, in the actual testing process, when conducting a detailed inspection of suspected damaged areas, the inspection scope is often appropriately expanded to the surrounding adjacent areas. Secondly, the error of this method is measured in grid units. As the grid division is refined, this spatial error decreases accordingly, making the advantage of this regional localization method more prominent. Although, this operation will inevitably increase the size of the training model and the time of sample data acquisition.

Ultimately, the experimental results indicate that the combination of frequency-domain features and time-domain features fully characterizes the variable cross-sectional geometric configuration and anisotropic material properties of wind turbine blades. In addition, the proposed impact source localization method could achieve a high localization accuracy with a single sensor, demonstrating its good applicability and application prospects.

## 5. Conclusions

This paper proposes a single-sensor impact source localization method based on EfficientNetV2. The method leverages the complex geometric configuration and the anisotropic material properties of wind turbine blades, thereby ensuring that AE signals from impact sources at different locations exhibit unique spatial coding characteristics. Combined with deep learning tools, it achieves high-precision regional localization. Some conclusions are drawn as follows:

(1) If features in both frequency and time domains are exploited, a 96.9% localization accuracy is achieved, and there is only one sample in the test set that produces a localization error be larger than one grid, demonstrating the effectiveness and accuracy of the proposed method.
(2) The method converts the impact source localization problem into a classification task. Its spatial localization accuracy depends on the granularity of region division—the finer the division, the smaller the localization error.
(3) The method achieves precise localization of impact sources on wind turbine blade structures without requiring prior knowledge of wave velocity, imposes no restrictions on material properties, and utilizes only a single sensor. It may provide an economic yet efficient monitoring solution for wind turbine blades.
(4) The current validation is limited to single impact events. Performance under simultaneous multi-impact events requires further investigation due to potential signal superposition effects. In addition, while the method imposes no material restrictions theoretically, its performance on other composite materials (e.g., carbon fiber blades) remains untested.

## Figures and Tables

**Table 1:** Parameters for EfficientNetV2-S model.

Stage	Operator	Stride	Channels	Layers	Kernel_Size
0	Conv	2	24	1	3
1	Fused-MBConv1	1	24	2	3
2	Fused-MBConv4	2	48	4	3
3	Fused-MBConv4	2	64	4	3
4	MBConv4	2	128	6	3
5	MBConv6	1	160	9	3
6	MBConv6	2	256	15	3
7	Conv&Pooling&FC	-	1280	1	1

**Table 2:** Localization accuracy and statistics of misclassified cases under different feature combinations.

Feature Combinations	Localization Accuracy	Statistics of Misclassified Cases	
		X_error_ ≤ 1 & Y_error_ ≤ 1	X_error_ > 1 or Y_error_ > 1
Real part only	68.8%	16	24
Imaginary part only	75.8%	11	20
Real + Imaginary parts	86.7%	8	9
Real + Imaginary parts + Time domain features	96.9%	3	1

**Table 3:** Results of localization errors.

Actual Region	Predicted Region	*X_error_*	*Y_error_*
1	3	2	0
54	55	1	0
55	63	0	1
56	64	0	1

## Data Availability

Dataset available on request from the authors.
